# Evaluation of a national universal coverage campaign of long-lasting insecticidal nets in a rural district in north-west Tanzania

**DOI:** 10.1186/1475-2875-11-273

**Published:** 2012-08-10

**Authors:** Philippa A West, Natacha Protopopoff, Mark W Rowland, Matthew J Kirby, Richard M Oxborough, Franklin W Mosha, Robert Malima, Immo Kleinschmidt

**Affiliations:** 1 Department of Infectious Disease Epidemiology, London School of Tropical Medicine and Hygiene, Keppel Street, London, UK; 2 Department of Disease Control, London School of Tropical Medicine and Hygiene, Keppel Street, London, UK; 3 National Institute for Medical Research, Amani Medical Research Centre, Muheza, Tanzania; 4 Kilimanjaro Christian Medical College, Tumaini University, Moshi, Tanzania; 5 MRC Tropical Epidemiology Group, London School of Tropical Medicine and Hygiene, Keppel Street, London, UK

**Keywords:** Malaria, Universal coverage, Vector control, Evaluation, LLIN, Tanzania

## Abstract

**Background:**

Insecticide-treated nets (ITN) are one of the most effective measures for preventing malaria. Mass distribution campaigns are being used to rapidly increase net coverage in at-risk populations. This study had two purposes: to evaluate the impact of a universal coverage campaign (UCC) of long-lasting insecticidal nets (LLINs) on LLIN ownership and usage, and to identify factors that may be associated with inadequate coverage.

**Methods:**

In 2011 two cross-sectional household surveys were conducted in 50 clusters in Muleba district, north-west Tanzania. Prior to the UCC 3,246 households were surveyed and 2,499 afterwards. Data on bed net ownership and usage, demographics of household members and household characteristics including factors related to socio-economic status were gathered, using an adapted version of the standard Malaria Indicator Survey. Specific questions relating to the UCC process were asked.

**Results:**

The proportion of households with at least one ITN increased from 62.6% (95% Confidence Interval (CI) = 60.9-64.2) before the UCC to 90.8% (95% CI = 89.0-92.3) afterwards. ITN usage in all residents rose from 40.8% to 55.7%. After the UCC 58.4% (95% CI = 54.7-62.1) of households had sufficient ITNs to cover all their sleeping places. Households with children under five years (OR = 2.4, 95% CI = 1.9-2.9) and small households (OR = 1.9, 95% CI = 1.5-2.4) were most likely to reach universal coverage. Poverty was not associated with net coverage. Eighty percent of households surveyed received LLINs from the campaign.

**Conclusions:**

The UCC in Muleba district of Tanzania was equitable, greatly improving LLIN ownership and, more moderately, usage. However, the goal of universal coverage in terms of the adequate provision of nets was not achieved. Multiple, continuous delivery systems and education activities are required to maintain and improve bed net ownership and usage.

## Background

Insecticide-treated nets (ITN) are one of the most effective measures for preventing malaria [1]. During the past decade a rapid scale-up of mosquito bed net coverage has been observed in sub-Saharan African countries [2], targeting first the most vulnerable populations, i.e., children under five and pregnant women [3], and more recently aiming to cover 80% of the population at risk for malaria in Africa [4]. To reach this goal, countries have implemented several net delivery systems including routine (free or subsidized) distribution of nets as part of health facilities' services, implemented by the public sector or through public and private partnership, and mass free distribution of nets during stand-alone campaigns or combined with immunization campaigns [5]. A combination of these strategies has been effective for scaling up net coverage whilst at the same time addressing concerns about equity in the provision of nets [6].

Tanzania has an extensive ITN national implementation plan based on over 25 years of experience [7,8] and supported by the Global Fund to Fight AIDS, Tuberculosis and Malaria (GFATM) and the USA President’s Malaria Initiative (PMI). The strategy is based on a combination of distribution mechanisms targeting different groups in the population: 1) starting in 2004 a public-private partnership continues to provide subsidized ITNs to pregnant women through discounted vouchers issued at antenatal clinics (TNVS) [9]; 2) between 2009 and 2010 a national mass campaign (U5CC) distributed 8.7 million long-lasting insecticidal nets (LLINs) free of charge to families with children under five years of age to quickly scale up ownership and usage in this group [10]; 3) in 2011 a Universal Coverage Campaign (UCC) distributed 17.6 million LLINs to ensure that all sleeping places are covered by an ITN and to increase ITN usage to 80% in the general population [11].

Muleba district in Kagera region (north-west Tanzania) received special attention due to its high malaria prevalence [12] and recurrent epidemics [13,14]. Since 2007, the district received yearly rounds of indoor residual spraying (IRS) using the pyrethroid lambdacyhalothrin (ICON®10CS, Syngenta, Basel, Switzerland). Spraying was carried out by the Research Triangle Institute (RTI) and funded by PMI. Net distribution has been ongoing as part of the national malaria strategy with the routine distribution of LLINs to pregnant women and under fives' campaign in July 2009 when 104,142 LLINs were issued to households with children under five years. In April 2011 the district, supported by Mennonite Economic Development Associates (MEDA), completed the UCC distributing 144,000 LLINs (Olyset, Sumitomo) to the population of Muleba.

This study used household surveys in Muleba district before and after the UCC to evaluate the campaign against its objectives, to determine its impact on LLIN ownership and usage, and to identify factors that may be associated with inadequate coverage.

## Methods

### Description of the study area

The study was carried out in 68 rural villages in Muleba district (1° 45’ S 31° 40’ E), in Kagera region of north-west Tanzania on the western shore of Lake Victoria. Muleba district (excluding islands) covers an area of 3,550 sq km and has a population of 487,878 people (Muleba District Office). The district is composed of 43 wards and 160 villages, the hamlet being the smallest administrative unit. In the study area there are on average 4.3 hamlets per village and 146 households per hamlet. The study area is situated at 1,100-1,600 m above sea level, includes 68,108 households and covers approximately two-thirds of Muleba district.

The Malaria Atlas Project estimated Muleba to have a 5-40% *Plasmodium falciparum* parasite rate [15]. Kagera region faced a major malaria epidemic at the end of 1997/beginning of 1998 [13]. Unverified information reports that Muleba also experienced outbreaks in 2006 [14] and 2010 (RTI unpublished data). According to the national malaria indicator survey 2007–2008, malaria prevalence was 41% in children under five in 2008 in Kagera region [12]. Malaria transmission occurs throughout the year in the district with two peaks occurring in December to January and June to July, following the short and long rainy seasons (District Medical Office unpublished data).

The UCC, which began in January 2011, consisted of three steps: 1) household registration: teams visited each household and issued coupons to be redeemed against LLINs, based on the number of permanent sleeping places not covered by ITNs, but with a maximum of two coupons per household; 2) LLIN distribution: householders attended the village distribution point in April 2011 in order to receive LLINs; 3) provision of information and assistance with hanging LLINs: Red Cross and community volunteers visited householders after nets were distributed. The organization of the distribution was the same as for the U5CC and is described in detail elsewhere [10].

### Study design

Baseline surveys, carried out as part of a cluster-randomized trial comparing the effects of the combined use of IRS and LLINs *versus* LLINs alone on malaria transmission, provided the data for this study. The trial area was divided into 50 clusters following the mapping and enumeration of every household with hand-held Global Positioning System devices (Garmin *e*trex legend H®, Garmin International Inc, USA). Each cluster was composed of several hamlets and comprised a minimum of 200 households. Household cross-sectional surveys were undertaken in each cluster in March and July 2011, starting one month before the beginning of the UCC and three months after the end of the UCC respectively. Households (100 per cluster in the pre-UCC and 80 in the post UCC survey) were randomly selected from each cluster. Households with children from six months to 14 years old were eligible for inclusion. Household heads or other resident adults were interviewed, after written informed consent had been sought. Data on bed net ownership and usage, demographics of household members, and other household characteristics including factors related to socio-economic status (SES) were gathered using an adapted version of the standard Malaria Indicator Survey [16]. Specific questions relating to the UCC process were asked in the second survey after the UCC.

### Statistical analysis

Data were collected by interviewers using Pendragon^TM^ Forms (Pendragon Corporation Software, Libertyville, USA) and Personal Digital Assistants (PDA), and then transferred into a Microsoft Access database (Microsoft Corporation, Redmond, USA). All statistical analysis was done in STATA 12 (STATAcorp, Texas, USA).

For the purpose of this analysis "treated nets" refers to pre-treated nets that are less than one year old and nets that have been re-treated within the last year, but not LLINs. "ITNs" is used to refer to LLINs and "treated nets".

The performance of the UCC campaign was evaluated by assessing the following indicators before and after the UCC:

**Net coverage indicators:** 1) the proportion of households with at least one ITN, and 2) the average number of ITNs per household.

**Intra-household saturation coverage indicators**[17]: 1) the proportion of households with adequate ITNs for every resident (at least one ITN for every two people as recommended by WHO [18]), and 2) the proportion of households owning enough ITNs to cover every sleeping place. The latter could only be calculated after the UCC, as data on sleeping places was only recorded in the post-UCC survey.

**Net usage indicators**: Proportion of all residents (usual residents and visitors), children under five, children six months to 14 years and pregnant women who are sleeping under an ITN the previous night.

Net ownership and usage data was based on direct observation of nets and the information given by the household respondent. The proportions sleeping under an ITN the previous night were calculated from the household register and the record of who slept under each net. Sleeping place was reported by the household respondent. Using logistic regression the associations between households owning enough ITNs for every sleeping place and the following explanatory variables were assessed: SES, children <5 in the household, household size, household crowding (number of residents per room), level of schooling of the household head, housing density (number of households per sq km in the cluster) and altitude (mean altitude for all the households in the cluster in m above sea level). All variables that were associated with the outcome with a p-value ≤0.2 were fitted in the multiple variable logistic regression model and retained if the adjusted p-value was ≤0.05. SES tertiles were created using Principle Component Analysis of the following household characteristics: number of rooms, household crowding, level of schooling of the household head, type of house (including floor, wall, and roof materials) and ownership of livestock, farmland, bicycles, mobile phones and radios.

The proportion of households successful at each step (registration, attending a distribution point, receiving LLINs, and the hang-up campaign) of the UCC campaign was analysed to evaluate the operational effectiveness of the campaign. Only basic information was collected on UCC education messages, sensitization and the hang-up campaign (which was ongoing at the time of the survey).

### Ethics approval

The trial was approved by the ethics review committees of the Kilimanjaro Christian Medical College, the National Institute for Medical Research Tanzania and the London School of Hygiene and Tropical Medicine (5814). Written informed consent was obtained from respondents.

## Results

Some 3,246 households (17,546 individuals) were included in the survey before the UCC campaign, and 2,499 households (13,766 individuals) were included in the post-UCC survey.

### Bed net coverage, intra-household saturation coverage and usage

The proportion of households with at least one ITN increased from 62.6% before the UCC to 90.8% afterwards (Table 1) and the mean number of ITNs owned per household almost doubled to 2.1. The proportion of nets that were LLINs was approximately 88% both before and after the UCC. After the UCC, 55.7% of all residents reported they slept under an ITN the previous night, 1.4-fold higher than before. Usage was highest in pregnant women and children under five years of age, but the increase in usage between surveys was highest in older children and adults. Overall 35.5% of households reported that all residents had slept under an ITN the previous night after the UCC (calculated from the household register and reported net use). The proportion of all ITNs that were used on the night before the survey was 81.2% (95% CI 79.6-82.7) before the UCC but was reduced to 63.8% (95% CI 62.2-65.3%) after the UCC. The most common reasons for non-use, in order of frequency, were: no sleeping place to cover with a net; not hung; too old or torn; being washed; no mosquitoes; do not know how to hang the net; usual sleeper not here; net used for other purpose.

**Table 1:** Net ownership, net type and ITN usage before and after the UCC

		**Before the UCC**		**After the UCC**	
		**%**	**[95% CI], (N)**	**%**	**[95% CI], (N)**
**Proportion of households with at least 1 ITN**		62.6	[60.9-64.2], (3246)	90.8	[89.0-92.3], (2499)
**Mean number of ITN owned per household**		1.2	[1.1-1.2], (3246)	2.1	[2.0-2.1], (2499)
**Proportion of nets by type**			(4032)		(5553)
* LLIN*		88.7	[87.3-89.9]	88.3	[87.2-89.4]
* Treated Nets*		4.1	[3.3-5.0]	7.5	[6.7-8.5]
* Untreated Nets*		7.2	[6.2-8.4]	4.2	[3.5-4.9]
**Proportion of residents sleeping under an ITN last night**					
* All residents*		40.8	[39.4-42.2], (17546)	55.7	[54.2-57.3], (13766)
* Children under 5 years of age*		56.5	[54.5-58.6], (3138)	63.3	[61.1-65.5], (2488)
* Pregnant Women*		55.3	[48.7-61.6], (228)	63.0	[56.5-69.0], (224)
* 6 months - 14 years*		42.5	[40.9-44.1], (9148)	56.1	[54.3-57.9], (7162)

Proportions, 95% Confidence intervals (CI) and the total number (N) are presented.

**Figure 1:**
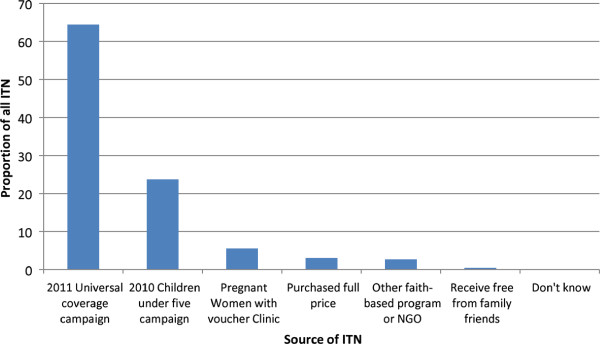
Source of ITNs in the post-UCC survey, n = 5,134.

After the UCC, 58.4% of households had at least one ITN per sleeping place (Table 2). Twenty percent of all households had more ITNs than sleeping places, 38.4% had the same number and 41.6% had too few ITNs for the number of sleeping places. When allowing for one ITN per two residents, a lower proportion (9.4%) had too many ITNs, but 35.2% of households had at least one ITN per two persons after the UCC, 2.7 times higher than before (before UCC: 13.2%, N = 3246, 95% CI 12.0-14.4) and the proportion varied widely between clusters from 9.8% to 53.1%. Data was collected on 5,553 bed nets after the UCC and 95.8% of these were ITNs. 64.5% of the ITNs were sourced from the UCC, 23.7% were from the 2010 U5CC and 5.5% from the pregnant women voucher scheme (Figure 1).

**Table 2:** Intra-household saturation coverage of ITNs after the UCC

	**1 ITN per sleeping place for all HHs (N = 2,493)**	**1 ITN per 2 residents for all HHs (N = 2,499)**	**1 ITN per sleeping place for HHs that received nets (N = 1,982)**
	**% [95% CI]**	**% [95% CI]**	**% [95% CI]**
**Households with:**			
Too few ITNs	41.6 [37.9-45.3]	64.8 [61.8-67.8]	35.2 [31.5-39.1]
Correct amount of ITNs	38.4 [36.2-40.7]	25.8 [24.0-27.8]	41.6 [39.1-44.2]
Too many ITNs	20.0 [17.5-22.8]	9.4 [7.9-11.0]	23.2 [20.3-26.3]
Intra-household saturation coverage of ITNs	58.4 [54.7-62.1]	35.2 [32.2-38.4]	64.8 [60.9,68.5]

The proportions of households (HH) and 95% confidence intervals (CI) are reported.

Households owning at least one net per sleeping place was independently associated with the presence of children under five years of age in the household and inversely associated with household size (Table 3). After adjusting for SES, cluster housing density and household crowding no longer showed an association. Elevation and schooling level of the household head were not associated with net ownership.

**Table 3:** Determinants of intra household saturation of ITNs

**Household Characteristic**		**Intra-household saturation**^**1**^**% (N)**	**Unadjusted**		**Adjusted**^**2**^	
			**OR [95% CI]**	**p - value**	**OR [95% CI]**	**p - value**
***HH Size (Number of residents)***						
* 2-4*		65.4 (784)				
* 5-12*		55.2 (1709)	0.65 [0.53-0.8]	<0.0001	0.53 [0.42-0.66]	<0.0001
***HH has individuals <5 years old***						
* No*		46.9 (825)				
* Yes*		64.1 (1668)	2.03 [1.68-2.44]	<0.0001	2.35 [1.94-2.85]	<0.0001
***3 percentiles of SES score***						
* Poorest*		61.0 (813)				
* Mid*		60.5 (823)	0.98 [0.78-1.22]	0.0261	1.14 [0.91-1.44]	0.2918
* Richest*		54.2 (802)	0.76 [0.6-0.95]		0.98 [0.78-1.24]	

Logistic regression allowing for clustering was used to produce the presented unadjusted and adjusted odds ratios (OR), 95% Confidence Intervals (CI) and p-values. ^1^Intra household saturation is defined here as one ITN or more per sleeping place in the household. ^2^Values are adjusted for household size and residents <5 years old in the household.

### Evaluation of the universal coverage campaign process

#### Step 1: household registration

**Figure 2:**
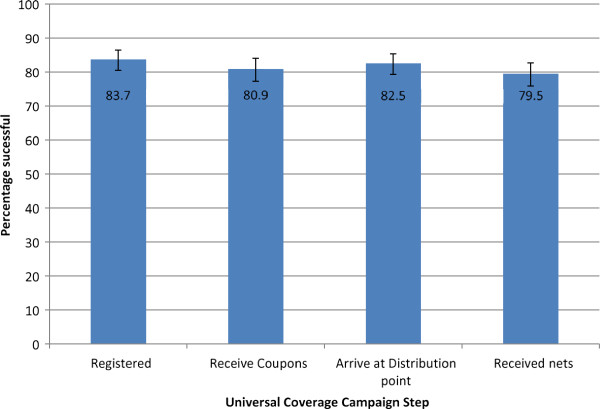
The proportion of all households that completed each step in the UCC process Proportion and 95% Confidence intervals are presented.

Of households in the survey, 83.7% (2091/2499) reported that they were registered during the UCC campaign (Figure 2). Nine households did not know if they were registered. Amongst the 399 households not registered, 39.6% claimed that the registration team did not come and 33.8% mentioned they were not at home during the registration day. Of registered households, 96.3% (2014) received coupons that would allow them to claim LLINs.

#### Step 2: arriving at the LLIN distribution point and receiving LLINs

Overall, 2,062 households (82.5%) reported that a household member attended the distribution point. Among those that were registered and received coupons, 97.5% (1,964/2,014) attended the distribution point. The most common reasons for registered households not attending the distribution point were: 22 forgot (27.9%), 15 had no time or means to go (19.0%) and 13 lost the coupons (16.5%). Ninety-eight households reported attending the distribution point even though they had not reported registering and having coupons.

Overall, 95.9% (1,977) of the 2,062 households that reported attending the distribution point received LLINs. Of the 85 that attended and did not receive LLINs, 31 were neither registered nor had received coupons, 18 were registered but had no coupons, 35 were registered and had received coupons (10 of which had lost the coupons), and one household reported having a coupon but not being registered. Reasons reported for not receiving LLINs at the distribution point included: 44 said they were refused nets (51.8%), 25 reported nets were not available (29.4%) and 10 said they lost the coupon (11.8%). In total, 1,986 households, 79.5% of all households surveyed, reported receiving LLINs from the distribution point, regardless of whether they reported attending.

#### Step 3: provision of information and assistance on hanging LLINs

Information on how to hang or use the nets was received at some point during the UCC by 69.3% of households. This increased to 74.5% in households that received nets. Some 5.8% of households reported receiving information from multiple sources. The community leaders or health workers were mentioned as a source of the information in 58.6% and 22.6% of responses, respectively.

Of all households that received nets, 64.6% had hung all the nets received, 14.8% had hung some but not all of the nets and 20.6% had not hung any of the nets. The proportion of households that had hung all the nets decreased as the number of nets received per household increased. Nets were usually hung by a household member (88.9%), but were sometimes hung by a village health assistant (8.8%) or another community member (2.0%).

## Discussion

The coverage of ITNs was increased greatly by the campaign with the proportion of households with at least one ITN increasing from 63% to 91%, which is high when compared to other mass distribution campaigns [5,17,19], and the mean number of ITNs per household almost doubled to 2.1. The proportion of nets that were LLINs remained constant. This was not due to misclassification as the percentage of nets that were LLINs was the same when considering only the nets that the fieldworkers observed, and the LLINs are easily differentiated as in this area the only LLINs are olyset nets. The main sources for the non LLINs after the UCC were: shops where they were purchased full price (42%), received from faith based programs or other NGOs (28%) or the antenatal voucher scheme (25%). The overwhelming majority of households that were registered also completed the remaining steps of the UCC, resulting in 79.5% of all households surveyed receiving LLINs. The attendance of non-registered households at the distribution point implies that households were keen to receive LLINs. Success rates for each step were as reported by household members up to two months afterwards and these may have differed from the true success rates.

The proportion of households with at least one ITN per two residents increased from 13% to 35%. Overall, 58% of households reported having enough ITNs for all sleeping places after the UCC. This is a more appropriate indicator for estimating intra-household coverage in this setting, as the average number of people per sleeping place was 2.4 (95% CI = 2.3-2.5). However, the stated aim of this UCC to provide one ITN per sleeping place was not achieved. A failing of the distribution was that 35% of households that received LLINs did not receive enough to cover all sleeping places. There were important variations between clusters; the proportion of households in a cluster that did not have enough nets for every sleeping place ranged from 31% to 85%. Low intra-household coverage was associated with larger household size and the absence of residents under five years old. The UCC limited the number of LLINs distributed to two per household which particularly affected ownership in large households and households with older children that had not benefitted from previous targeted net distribution campaigns [10]. Another problem was the registration procedure with 16% of households reporting not being registered. However, considering the size and speed of the campaign this is a relatively good performance and the coverage attained was similar to what has been achieved by indoor residual spraying campaigns [20,21]. It was reported that non-registration was due to either the registration team not visiting the house or that the household owners were not at home. The proportion of houses being registered could be improved by the team making repeated visits or spending more time finding households.

Another challenge was the estimation of net needs at household level during the registration process, which could explain why 23% of households have more nets than sleeping places, with variation from 6% to 45% according to cluster. The household needs for nets were based on direct observation or verbal report from the beneficiaries of how many nets were owned. The over-estimation could be explained by people exaggerating their need for nets. The present study illustrates that even in a well-planned and implemented mass net distribution campaign it is difficult to appropriately evaluate the needs at household level, especially when moderate net coverage has been already achieved and when accurate numbers of pre-existing nets are not known. In the present study 73% of all sleeping places could be covered by an ITN, however with the number of ITNs observed there would be enough to cover 83% of all sleeping places, if the allocation had been optimal. This reinforces Kilian and colleagues [17] call to revise the allocation procedure of nets at the household level in order to increase intra-household coverage and reach universal coverage. Continuous distribution of LLINs to pregnant women during antenatal consultation will top up net coverage and replace worn out nets in households with women of child-bearing age. However an additional continuous distribution mechanism needs to be set up for other households.

Household SES was not associated with households having enough nets, suggesting that the UCC was equitable. This is an important achievement of the UCC as it is usually the poorest that are most vulnerable to malaria and yet often benefit least from public health interventions. Mass distribution campaigns have previously been reported to be equitable [22,23] and this study provides further evidence to support that claim.

Although the number of nets owned per household almost doubled after the UCC, a more modest increase in usage (from 41% to 56%) was reported. This would not be high enough to gain the full benefit of the “mass effect” from high ITN usage in the community [24,25]. In households with enough nets for every sleeping place only 44% reported that all residents in the household slept under a net the previous night, suggesting that net usage could be improved. The percentage of all ITNs used was slightly lower in households with enough ITNs for all sleeping places compared to those with insufficient nets (62% and 68% respectively). A similar gap between ownership and usage has been reported from other studies [10,26]. The increase in usage is likely to be a consequence of the increased availability of ITNs at the household level due to the UCC, however the effect of seasonality cannot be accounted for in this study. The level of net usage can be affected by a variety of factors including temperature, humidity, season and mosquito density [27], and thus reported usage levels could have been higher or lower if the post-UCC survey was conducted at a different time. ITN usage was highest in the children under five and pregnant women (63% in both) who were targeted in previous net distribution strategies.

Hang-up campaigns are important to convert ownership into usage [23]. Sixty percent of those receiving LLINs from the UCC had hung all of the nets. Nearly 90% of the households with nets hung reported that they were hung by household members; this either means that they did not need help to hang their net from the hang-up campaign volunteers or that the volunteers were not very active as they were supposed to assist with hanging the nets.

Only households with residents between the ages of six months and 14 years were included in the survey as this was an inclusion criterion for the randomized controlled trial. ITN ownership and use is therefore likely to be higher in the study population than in the population as a whole as these households would have benefited from previous net distributions and may have better knowledge about the benefits of ITNs. Community leaders were often present during household interviews and this potentially may have led interviewees to report more positively about the UCC as it is likely that the leaders were involved in the UCC. However this influence should be limited as the interviewer was independent from the UCC team and any over-estimation of net ownership and usage was minimized as nets were observed by the interviewer and nets that had never been hung could not be reported as used.

## Conclusion

The UCC in Muleba was successful and marks a significant advance on the progression in ITN policy from targeted provision to mass distribution to all [4,18] and showed that a free mass distribution can rapidly increase ITN ownership in an area that had previously received targeted distribution to pregnant women and children under five years of age. The UCC campaign is shown to be equitable, with no evidence that poorer households benefited less. However, despite the significant gains achieved the goal of universal coverage was not reached due to inadequate allocation of nets at the household level and low rates of usage. Additional delivery methods to supplement the existing antenatal distribution to pregnant women would be required to reach and maintain universal coverage. This will necessitate strategic planning to address the difficult issue of maintaining high ownership and usage of LLINs in rural Tanzania.

## Competing interests

The authors declare that they have no competing interests.

## Authors’ contributions

PW analysed the data, drafted the paper and contributed to the data collection and study design. NP provided crucial input into the data analysis and writing the manuscript and contributed to the study design and data collection. IK provided major input into the data analysis and writing the manuscript and contributed to the study design. MR contributed to the study design and improved the manuscript. MK and RO contributed to the overall project study design and provided comments on the manuscript. RM and FM contributed to the overall project study design. All authors read and approved the final manuscript.
